# Supplementary material for: Silver Nanoparticles Embedded on Reduced Graphene Oxide@Copper Oxide Nanocomposite for High Performance Supercapacitor Applications
Source: Materials (Basel). 2021 Sep 3;14(17):5032. doi: 10.3390/ma14175032 (PMC8434351; doi:10.3390/ma14175032)
Supplement: Supplementary file 1 [file materials-14-05032-s001.zip › materials-1336671-Supplementary.pdf]

materials

MDPI

Article

Silver Nanoparticles Embedded on Reduced Graphene Oxide@Copper Oxide Nanocomposite for High Performance Supercapacitor Applications

Akhalakur Rahman Ansari[1,2], Sajid Ali Ansari[3], Nazish Parveen[4], Mohammad Omaish Ansari[2] and Zurina Osman[1,5*]

[1] Department of Physics, Faculty of Science, Universiti Malaya, 50603, Kuala Lumpur, Malaysia; akhalakur-kau@gmail.com

[2] Center of Nanotechnology, King Abdulaziz University, Jeddah, 21589, Saudi Arabia; omishchem@gmail.com

[3] Department of Physics, College of Science, King Faisal University, P.O. Box, 400, Hofuf, Al-Ahsa, 31982, Arabia;sansari@ kfu.edu.sa

[4] Department of Chemistry, College of Science, King Faisal University, P.O. Box, 380, Hofuf, Al-Ahsa, 31982, Saudi Arabia;nislam@kfu.edu.sa

[5] Centre for Ionics Universiti Malaya, Universiti Malaya, 50603, Kuala Lumpur, Malaysia; zurina-osman@um.edu.my

* Correspondence: zurinaosman@um.edu.my; Tel.: +603-79674206/4288

Citation: Ansari, A.R.; Ansari, S.A.; Parveen, N.; Ansari, M.O.; Osman, Z. Electrochemical properties of Ag nanoparticles embedded on a composite of reduced graphene oxide and CuO nanosheets. *Materials* **2021**, *14*, 5032. https://doi.org/10.3390/ma14175032

Received: 27 July 2021
Accepted: 30 August 2021
Published: 3 September 2021

Publisher's Note: MDPI stays neutral with regard to jurisdictional claims in published maps and institutional affiliations.

Copyright: © 2021 by the authors. Submitted for possible open access publication under the terms and conditions of the Creative Commons Attribution (CC BY) license (http://creativecommons.org/licenses/by/4.0/).

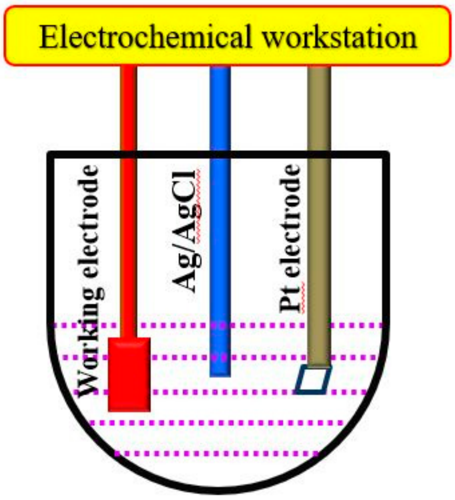

Figure S1. Schematic diagram of the electrochemical cell.

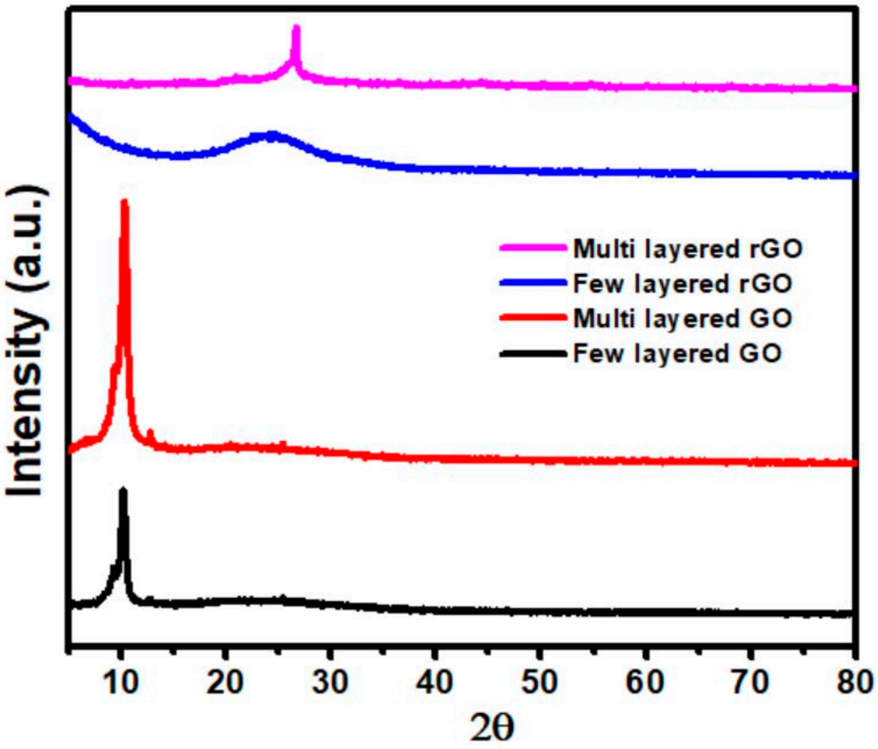

Figure S2. XRD pattern of few layered and multi layered GO and rGO.
